# Assessment of cerebral circulation in a porcine model of intravenously given E. coli induced fulminant sepsis

**DOI:** 10.1186/s12871-017-0389-0

**Published:** 2017-07-24

**Authors:** Levente Molnár, Norbert Németh, Mariann Berhés, Endre Hajdú, Lóránd Papp, Ábel Molnár, Judit Szabó, Ádám Deák, Béla Fülesdi

**Affiliations:** 1 Department of Anesthesiology and Intensive Care, University of Debrecen, Faculty of Medicine, Nagyerdei krt. 98, Debrecen, H 4032 Hungary; 2 Department of Operative Techniques and Surgical Research, University of Debrecen, Faculty of Medicine, Debrecen, Hungary; 3 Department of Medical Microbiology, University of Debrecen, Faculty of Medicine, Debrecen, Hungary; 4 Outcomes Research Consortium, Cleveland, USA

**Keywords:** E coli, Experimental sepsis, PiCCo monitoring, Transcranial Doppler, Cerebral hemodynamics

## Abstract

**Background:**

The aim of the present work was to assess cerebral hemodynamic changes in a porcine model of E.coli induced fulminant sepsis.

**Methods:**

Nineteen healthy female Hungahib pigs, 10–12 weeks old, randomly assigned into two groups: Control (*n* = 9) or Septic Group (*n* = 10). In the *Sepsis group Escherichia coli* culture suspended in physiological saline was intravenously administrated in a continuously increasing manner according to the following protocol: 2 ml of bacterial culture suspended in physiological saline was injected in the first 30 min, then 4 ml of bacterial culture was administered within 30 min, followed by infusion of 32 ml bacterial culture for 2 h. Control animals received identical amount of saline infusion. Systemic hemodynamic parameters were assessed by PiCCo monitoring, and cerebral hemodynamics by transcranial Doppler sonography (transorbital approach) in both groups.

**Results:**

*In control animals*, systemic hemodynamic variables and cerebral blood flow velocities and pulsatility indices were relatively stable during the entire procedure. *In septic animals* shock developed in 165 (IQR: 60–255) minutes after starting the injection of E.coli solution. Blood pressure values gradully decreased, whereas pulse rate increased. A decrease in cardiac index, an increased systemic vascular resistance, and an increased stroke volume variation were observed. Mean cerebral blood flow velocity in the middle cerebral artery did not change during the procedure, but pulsatility index significantly increased.

**Conclusions:**

There is vasoconstriction at the level of the cerebral arterioles in the early phase of experimental sepsis that overwhelmes autoregulatory response. These results may serve as additional pathophysiological information on the cerebral hemodynamic changes occurring during the septic process and may contribute to a better understanding of the pathomechanism of septic encephalopathy.

## Background

The central nervous system is one of the first organs to be affected in the early stage of septic inflammatory processes; it is estimated that consciousness differences of various severity may appear in up to 70% of septic cases [1–3]. The pathomechanism of sepsis-associated encephalopathy is complex, microcirculatory alterations, disturbance of cerebral autoregulation, blood–brain barrier damage, branched chain/aromatic amino acid inbalance and the direct effect of the inflammatory process (e.g. free radicals, oxydative stress, cytokines, excitotoxicity apoptosis) on glial cells may play a determining role [4].

The exact role of cerebral macro-and microcirculation in the development of sepsis-associated encephalopathy is a debated issue. In experimental sepsis, cerebral blood flow (CBF) has been reported to be unchanged [5, 6], decreased [7], or even increased [8, 9], depending on the experimental model, the species of the study animals and the progression and severity of sepsis. Human studies have found decreased CBF in patients with sepsis, which suggests a possible causative role of cerebral ischemia in the development of neurological symptoms [10, 11]. However, there have been reports on normal CBF measured by transcranial Doppler sonography (TCD) [12]. Previous studies have reported on normal cerebral autoregulation and vasoreactivity in septic patients [13]. Cerebral vasoreactivity to various stimuli has been found to be unchanged or decreased in patients with sepsis [14–16]. There are also observations suggesting that the involvement of the cerebral microvasculature may be different in different severities of the septic process [16]. The majority of the previous observations were performed in humans or in animals with hyperdynamic sepsis and no information could be found on the cerebral blood flow and cerebral vasoreactivity in hypodynamic sepsis models.

In view of the previous observations, in the present study we tested the hypothesis whether or not cerebral autoregulation is affected in a porcine model of intravenously given E.coli-induced experimental sepsis.

We intended to answer the following study questions:

1. What is the impact of the alteration of systemic hemodynamic parameters on cerebral blood flow velocity during the development of fulminant septic shock?
2. Can we detect affected cerebral autoregulation during developing fulminant sepsis?

## Methods

### Experimental animals and protocol

Nineteen healthy female Hungahib pigs, 10-12 weeks old, were involved in the experiment and randomly assigned into two groups: Control (*n* = 9) or Septic Group (*n* = 10). The weight and length of the animals in the two groups were: weight control 19 (16.7-20.2) kg vs. weight septic: 18.5 (18.8-20.0) kg, *p* = 1.0; length control: 92 (83.5-97.2) cm vs. length septic: 92.0 (85–100) cm, *p* = 0.96. According to the original protocol the inclusion of 20 animals was planned (ten pigs in each group). However, one animal was injured during transport and was thus not included.

The experiments were approved and registered by the University of Debrecen Committee of Animal Welfare (permission Nr.: 21/2013. UD CAW), in accordance with the Hungarian Animal Protection Act Law XVIII/1998 and the Ordinance 40/2013. (II.14.) of the Hungarian Government and EU directive.

In the *Sepsis group Escherichia coli* culture (2.5 × 10^5^/ml; strain: ATCC 25922, Department of Medical Microbiology, University of Debrecen) suspended in physiological saline (Api NaCl 0.85 Medium and suspension medium, bioMérieux SA, Lyon, France) was intravenously administrated in a continuously increasing manner according to the following protocol: 2 ml of bacterial culture suspended in physiological saline was injected in the first 30 min, then 4 ml of bacterial culture was administered within 30 min, followed by infusion of 32 ml bacterial culture for 2 h. Thus, a total of 9.5 × 10^6^ E.coli was administered within 3 h. According to our laboratory tests, at 3 h after suspending the E.coli, the number of the living bacteria remained stable.

Subjects of the *Sepsis group* were examined until they died as a cause of the fatal infection. In the *Control Group* infusion was administered in a similar volume to the *Septic Group* of isotonic saline solution and no further intervention was carried out on them. Each individual of this group was followed for 8 h (if the animals had not died earlier) and at the end of the experimental period the animals were over-anaesthetized.

The study was carried out under general anesthesia maintained by giving intramuscular ketamine (15 mg/kg) and xylazine (1 mg/kg) throughout the experiment. The depth of anesthesia was assessed by blood pressure and heart rate changes to noxious stimuli, and was adjusted if necessary by intermittent boluses of ketamine and xylazine. Both in the Sepsis and Control groups inferior tracheostomy was performed and an endotracheal tube was inserted for supported ventilation. Pressure support mechanical ventilation (Airox Legendair Ventilator, PAU Cedex France) was used. Mechanical ventilation was adjusted to secure a PaO_2_ of 100–130 mmHg and PaCO_2_ of 35–45 mmHg.

Besides physiological saline infusion the animals were not given anticoagulants or any further medication during the experiment. The temperature of the operating room was set to approximately 25 °C and a 37 °C heating pad was placed under the animals to maintain body core temperature above 37 °C. A suprapubic cystostomy catheter was placed to ensure urinary drainage.

### Hemodynamic measurements

The left external jugular vein and the left femoral artery were surgically prepared and cannulated for invasive hemodynamic measurements, and blood sampling. After all surgical interventions had been completed, a 1-h long stabilization period was allowed before the beginning of the experimental protocol. Cardiac output (CO) and systemic vascular resistance index (SVRI) were assessed by thermodilution using a 4F, 8 cm PiCCO®-Catheter (Pulsion Medical Systems AG, Munich, Germany) with the injection of 10 (x) mL of cold saline each hour. Heart rate (HR (1/min)) and mean arterial pressure (MAP (mmHg)) were monitored invasively through the femoral artery catheter. The Meeh’s formula was used for calculation of body surface area in pigs (BSA = 8.58xBW).

### Monitoring cerebral blood flow parameters

The animal subjects were placed in the prone position and Transcranial Doppler ultrasound measurements were carried out using a Rimed Digilite Transcranial Doppler sonograph (Rimed Ltd., Raanana, Israel). A 2 MHz probe was placed on the left transorbital window for insonation, while sample volume, gain, power and angle of the ultrasound probe were kept constant during the whole investigation procedure. Signals detected from the middle cerebral artery between the depths of 15 to 25 mm were assessed. Each measurement was repeated three consecutive times and the highest value was taken into account for our analysis. Systolic, diastolic and mean cerebral blood flow velocities (cm/s) were registered, while pulsatility indices (PI) were calculated by the device. Measurements were performed at resting state = TR, and every hour following the start of the injection of the E coli or isotonic saline during the experiment = T60-T420. Resting measurements were performed before starting suspension or saline, (indicated as RS). At 60 min (indicated as T60) the injection of 2 + 4 ml bacterial culture/saline was completed, at 120 min (indicated as T120) and at 180 min (T180) additional 16 ml cultures or saline solutions were infused.”

### Blood gas monitoring

Arterial blood samples were collected each hour starting prior to the administration of bacterial suspension or isotonic saline. Samples were used to measure pH, PO_2_, PCO_2_, hemoglobin and base excess using a blood gas analyzer (GEM Premier 3500 Blood Gas Analyser, Werfen International, Milan, Italy).

### Statistical analysis

Statistical analysis was performed using SPSS 19.0 (SPSS, Chicago, IL). Kolgomorow-Smirnov test was used to verify the normality of the distribution of continuous variables. As the majority of the parameters did not show normal distributions, data are presented as medians and interquartile ranges and parameters were compared by the appropriate non-parametric tests (Mann–Whitney Rank Sum Test).

The analysis of the treatment effect on the different parameters within the groups occurred by using repeated measures ANOVA. To calculate the correlation between different variables Spearman correlation was used. A *p* < 0.05 was defined as statistically significant difference.

## Results

Of the ten septic animals, three died after 3 h, another three at 5 h and one after 6 h after the E.coli infusion was started. Most probably due to seasonal variation of the animal experiments, three animals originally randomized to control group were excluded from the analysis, because the intial PiCCo measurements showed unusually increased baseline SVRI values. These animals were all included in the winter period whereas all the others during spring or early fall. For sake of clarity we deleted their data from the analysis and repeated the measurements in additional three animals in spring 2017. Data presented here are from this analysis. Resting systemic and cerebral hemodynamic parameters and blood gas analysis results of the control and septic groups are summarized in Table 1.

**Table 1:** Systemic and cerebral hemodynamic parameters at resting state in control and septic animals

Parameters	Control	Septic	*P*–value
BP Sys (mmHg)	120.0 (116.0–134.3)	124.0 (119.0–140.0)	0.73
BP diast (mmHg)	92.0 (89.0–99.2)	91.0 (85.0–100.0)	0.44
MAP (mmHg)	100.0 (96.2–108.8)	102.3 (96.3–113.3)	0.91
Pulse rate (1/min)	89.0 (72.2–94.0)	86.0 (77.0–95.0)	0.71
PaO_2_ (mmHg)	82.0 (77.5–92.7)	89.5 (84.0–97.0)	0.47
PaCO_2_ (mmHg)	43.0 (41.2–48.2)	43.5 (40.0–46.0)	0.48
pH	7.44 (7.41–7.45)	7.44 (7.43–7.45)	0.71
CI (l/min/m^2^)	2.2 (1.9–3.1)	2.3 (1.8–3.0)	0.63
SVRI (dyn.sec.cm^−5^. m^2^)	2823 (2478–3849)	2658 (2139–3301)	0.25
GEDI (ml/m^2^)	428 (346–470)	415.0 (336.0–636.0)	0.42
SVV (%)	13.0 (10.2–14.2)	10.0 (7.0–13.0)	0.37
GEF (%)	28.0 (23.0–33.5)	26.5 (22.0–28.0)	0.46
EVLWI (ml/kg)	15.0 (9.7–16.5)	12.0 (10.0–17.0)	0.88
TCD systolic (cm/s)	27.0 (26.0–36.5)	32.0 (26.0–36.0)	0.72
TCD diastolic (cm/s)	14.0 (11.7–16.2)	18.5 (14.0–20.0)	0.31
TCD mean (cm/s)	20.0 (17.2–23.2)	23.0 (17.0–24.0)	0.59
TCD PI	0.67 (0.63–0.78)	0.64 (0.55–0.67)	0.20

Medians and interquartile ranges are presented

*In control animals*, hemodynamic variables were relatively stable during the entire procedure. It should be noted that there was a slight but inconsistent decrease in diastolic blood pressure and in mean arterial pressure. Hemodynamic parameters as measured by PiCCo monitoring did not change significantly during the procedure. Similarly to blood gas parameters and pH. Transcranial Doppler measurements revealed constant cerebral blood flow velocities within the middle cerebral arteries with unchanged pulsatility indices (Table 2).

**Table 2:** Systemic and cerebral hemodynamic parameters and blood gas values measured in control animals

	Time point of measurement								
Parameters	TR	T60	T120	T180	T240	T300	T360	T420	*p*–value
BP Sys (mmHg)	120 (116–134.2)	117 (109–7–130.2)	120 (93.7–130.5)	123 (103–130.7)	119 (98.2–125.7)	118 (116.5–130)	130 (123.3–133.7)	118 (118–130)	0.09
BP diast (mmHg)	92 (89.5–99.2)	89 (84.7–93.2)	89 (84.7–93.2)	94 (82.2–97.0)	86 (81.5–91.5)	80.5 (64–96.5)	84.0 (77.2–85.5)	86 (83.0–87.5)	0.007
MAP (mmHg)	100 (96.2–108.1)	102 (90.7–105.8)	96 (90–108.5)	98 (84.2–102.5)	94 (82.2–105.7)	94.6 (90.6–100.2)	98.0 (97.2–102.2)	94 (91–100.5)	0.09
Pulse rate (1/min)	89 (72.2–94)	89 (73.2–95)	86 (79–98)	88 (77.2–96.2)	83.5 (78–92)	82 (79–90.2)	92 (84.5–93.5)	82 (81.2–85)	0.15
PaO_2_ (mmHg)	82 (77.5–92.7)	100 (83–113.5)	89 (76–114)	92 (81.5–100.2)	82 (74.7–89.7)	81 (77.2–106.5)	79 (75.2–99.2)	76 (76–77.1)	0.3
PaCO_2_ (mmHg)	43 (41.2–48.2)	38 (25.7–44)	40 (30.5–45.2)	42 (29–44.5)	45 (39–48.2)	38 (32–47)	35 (29–47)	43.5 (39–48)	0.07
pH	7.44 (7.41–7.45)	7.43 (7.41–7.55)	7.43 (7.39–7.52)	7.42 (7.38–7.45)	7.38 (7.36–7.43)	7.41 (7.35–7.44)	7.43 (7.36–7.46)	7.36 (7.34–7.38)	0.15
CI (l/min/m^2^)	2.2 (1.9–3.1)	2.5 (1.9–2.9)	2.2 (1.3–2.8)	2.1 (1.6–2.6)	2.2 (1.8–2.7)	2.2 (1.5–2.7)	1.8 (1.5–2.3)	2.1 (1.6–2.2)	0.08
SVRI (dyn.sec.cm^−5^. m^2^)	2823 (2478–3849)	2990 (2550–3916)	3227 (2731–4427)	3182 (2580–4677)	3088 (2813–4337)	3909 (3264–5328)	4340 (3626–4640)	3705 (3669–4387)	0.37
GEDI (ml/m^2^)	428 (346–470)	426 (342–524)	448 (329–480)	428 (346–470)	459 (358–482)	402 (334–499)	373 (244–388)	350 (230–381)	0.77
SVV (%)	13 (10.3–14.3)	14 (11–15)	19 (12.7–20.7)	15 (13.3–18.3)	17.5 (13–19)	19 (13.3–20.7)	15 (14.7–16.5)	18 (12.7–18)	0.27
GEF (%)	28 (23–33.5)	27 (22.5–31.5)	25 (21.7–28.7)	24.5 (22–27.5)	24.5– (23.5–26)	29 (24.7–29.3)	27 (24.7–28.2)	27 (25.5–27.3)	0.16
EVLWI (ml/kg)	15 (9.7–16.7)	14 (9.5–17.5)	16 (11–18.7)	17.5 (11.5–20.5)	15 (9–16.8)	12 (8.3–15.8)	13 (8.5–16)	13 (8.5–16.8)	0.22
TCD systolic (cm/s)	27 (26–36.5)	28 (26–36.5)	30 (23–39.2)	28 (23.7–35.5)	30 (24.5–33)	40 (33.2–45.2)	33 (33–34.5)	36 (34.5–36)	0.89
TCD diastolic (cm/s)	14 (11.7–16.2)	13 (10.5–16)	12 (10–17.7)	11 (10–16)	13 (9.7–14.7)	15 (11.2–18.2)	15 (13.5–15.2)	15 (12.7–21.2)	0.91
TCD mean (cm/s)	20 (17.2–23.2)	18 (16–20.5)	19 (16–24.5)	17 (15–21)	19 (15–20.8)	25 (19.7–28.7)	21 (20.2–21.7)	21 (21–25.5)	0.89
TCD PI	0.67 (0.64–0.78)	0.83 (0.71–0.88)	0.91 (0.63–1.09)	0.93 (0.67–1.03)	0.9 (0.73–1.02)	0.93 (0.85–1.01)	0.92 (0.87–0.93)	0.89 (0.61–0.99)	0.15

Medians and IQRs are presented. TR = resting state, T60–T420 = minutes after starting the injection of the E coli suspension

*In septic animals* shock developed in 165 (IQR: 60–255) minutes after starting the injection of E.coli solution. The change of the different parameters in this animal group is summarized in Table 3. Blood pressure values gradually decreased, whereas pulse rate increased in the pigs after injection of E.coli. Flow parameters indicated a decrease in cardiac index along with an increased systemic vascular resistance (SVRI). Additionally, stroke volume variation (SVV) showed a statistically significant increase with a non-significant decrease in global end-diastolic index (volume parameters). Extravascular lung water index increased, while global ejection fraction decreased during the procedure. As a consequence of the developing shock, pH values moved toward acidosis during the procedure with a slight but statistically insignificant increase in PaCO_2_ values.

**Table 3:** Systemic and cerebral hemodynamic parameters and blood gas values measured in septic animals

	Time point of measurement								
Parameters	TR *n* = 10	T60 *n* = 10	T120 *n* = 10	T180 *n* = 7	T240 *n* = 7	T300 *n* = 4	T360 *n* = 3	T420 *n* = 2	*p*–value
BP Sys (mmHg)	124.0 (119.0–140.0)	125.5 (105.0–130.0)	125.0 (77.0–143.2)	126.0 (110.5–137.0)	106.0 (86.0–113.0)	76.0 (70.2–114.5)	82.0 (65.5–118.0)	80.0 (72.5–116.0)	<0.001
BP diast (mmHg)	91.0 (85.0–100.0)	92.0 (70.0–100.0)	95.0 (56.0–111.5)	98.0 (78.5–108.5)	75.5 (63.0–90.0)	54.0 (49.2–84.5)	55.0 (46.7–91.7)	54.0 (43.5–90.7)	<0.001
MAP (mmHg)	102.3 (96.3–113.3)	103.2 (81.7–115.0)	105.0 (62.8–122.0)	107.5 (89.2–118.8)	85.6 (70.6–97.7)	60.6 (56.4–94.5)	64.0 (53.0–100.5)	62.7 (53.2–99.2)	<0.001
Pulse rate (1/min)	86.0 (77.0–95.0)	86.5 (80.0–96.0)	96.0 (79.7–98.5)	107.0 (92.0–139.5)	110.5 (102.0–125.0)	105.0 (84.5–122.7)	134.0 (111.5–162.5)	135.0 (110.2–140.2)	<0.001
PaO_2_ (mmHg)	89.5 (84.0–97.0)	86.0 (79.7–101.7)	71.0 (58.0–86.7)	70.0 (65.0–97)	78.0 (56.5–122.0)	87.5 (50.0–125.0)	118.0 (91.0–145.0)	55.0 (49.0–62.5)	0.18
PaCO_2_ (mmHg)	43.5 (40.0–46.0)	43.0 (35.2–43.2)	43.0 (36.7–45.0)	43.0 (38.0–46.2)	42.0 (38.0–50.0)	45.5 (41.0–50.0)	47.5 39.0–56.0)	48.0 (38.2–56.0)	0.72
pH	7.44 (7.43–7.45)	7.42 (7.39–7.48)	7.38 (7.36–7.43)	7.36 (7.31–7.39)	7.3 (7.22–7.37)	7.3 (7.24–7.36)	7.16 (7.13–7.2)	7.22 (7.17–7.32)	<0.001
CI (l/min/m^2^)	2.3 (1.8–3.0)	2.3 (1.8–3.3)	2.0 (1.6–2.6)	1.7 (1.5–2.3)	2.7 (1.7–3.1)	1.7 (1.1–1.7)	1.1 (1.0–1.7)	1.6 (1.3–1.8)	<0.001
SVRI (dyn.sec.cm^–5^. m^2^)	2658 (2139–3301)	3315 (2405–4122)	3331 (2729–4139)	4150 (3499–4504)	3254 (2061–5990)	4529 (4201–5254)	5687 (4724–5795)	4585 (1654–5519)	0.01
GEDI (ml/m^2^)	415.0 (336.0–636.0)	460.5 (387.5–646.0)	393.5 (317–561.5)	373.0 (227.2–534.0)	373.0 (222.2–403.7)	332.5 (219.0–474.0)	223.0(166.0–453.2)	254.0 (167.0–461.0	0.326
SVV (%)	10.0 (7.0–13.0)	8.0 (7.0–11.5)	11.0 (9.5–12.7)	17.0 (10.5–21.5)	14.0 (9.5–15.5)	20.5 (10.0–26.0)	18.0 (14.2–23.2)	14.0 (14.0–22.2)	0.017
GEF (%)	26.5 (22.0–28.0)	21.0 (20.0–26.2)	25.0 (19.0–27.0)	17.5 (14.5–25.0)	20.0 (17.7–21.5)	17.5 (12.0–20.0)	13.0 (10.7–17.5)	19.0 (14.5–21.5)	<0.001
EVLWI (ml/kg)	12.0 (10.0–17.0)	15.5 (11.0–23.0)	10.0 (8.2–18.0)	15.5 (7.0–23.5)	10.5 (6.0–15.0)	15.0 (7.0–22.0)	14.0 (8.0–26.0)	20.0 (8.7–27.5)	0.02
TCD systolic (cm/s)	32.0 (26.0–36.0)	33.5 (31.0–38.0)	38.0 (29.7–39.5)	32.5 (27.5–35.5)	38.0 (24.5–43.7)	29.0 (28.0–37.5)	26.6 (24.5–39.0)	29.0 (23.7–40.2)	0.933
TCD diastolic (cm/s)	18.5 (14.0–20.0)	17.5 (17.0–21.0)	18.0 (10.7–19.0)	12.5 (9.0–15.0)	11.0 (9.0–18.7)	9.0 (8.0–12.5)	6.0 (5.0–9.5)	6.0 (6.0–12.7)	<0.001
TCD mean (cm/s)	23.0 (17.0–24.0)	23.0 (21.0–28.0)	23.0 (18.2–26.5)	17.5 (17.0–21.0)	18.0 (15.0–28.2)	18.0 (15.0–18.0)	15.0 (11.5–19.5)	19.0 (13.0–19.7)	0.117
TCD PI	0.64 (0.55–0.67)	0.67 (0.57–0.78)	0.9 (0.62–1.0)	0.9 (0.86–1.4)	0.95 (0.86–1.15)	1.2 (1.07–1.58)	1.7 (1.16–2.0)	1.4 (0.88–1.88)	<0.001

Medians and IQRs are presented. TR = resting state, T60–T420 = minutes after starting the injection of the E coli suspension

### Transcranial Doppler measurements

All transcranial Doppler parameters were stable during the experimental procedures in control animals. Systolic and mean velocities within the middle cerebral arteries did not show statistically significant changes during the development of septic shock. It is worth mentioning, however, that there was a tendency toward decreasing mean blood flow velocities. A gradual decrease in MCA diastolic velocities as well as a significant increase in pulsatility indices were observed.

**Fig. 1:**
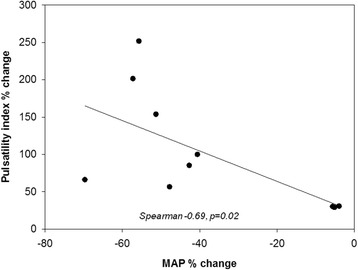
Relationship between percentual change in the mean arterial blood pressure (MAP%) and percentual change of the pulsatility index (PI%) in septic animals

In septic animals, a statistically significant relationship was found between the percentual decrease of the mean arterial blood pressure and the percentual increase of the pulsatility index, i.e. the greater the decline in the blood pressure was, the higher increases in pulsatility indices were observed (Fig. 1). There was no correlation between the percentual change of the mean arterial blood pressure and the middle cerebral artery mean blood flow velocity percentual changes (Spearman coefficient of correlation: 0.26, *p* = 0.44). Similarly, no relationship was found between percentual changes of the MCA mean blood flow velocity and percentual changes of the pulsatility index (Spearman coefficient of correlation: 0.01, *p* = 0.97).

## Discussion

In the present study we assessed whether development of an E-coli-induced experimental septic shock results in changes in cerebral blood flow velocity in pigs. We found that despite the gradual decrease in the systemic blood pressure during the development of septic shock, middle cerebral artery mean blood flow velocity remained relatively stable during the procedure. Although we observed a slight decrease in middle cerebral artery mean blood flow velocity, the changes did not reach the level of statistical significance.

Based on our results in septic animals, one may conclude that cerebral autoregulation is intact, because mean blood flow velocity (that is proportional to changes in cerebral blood flow) was found to be relatively stable during the course of the developing septic shock. However, the increase in the pulsatility index suggests a different pathomechanism.

Cerebral autoregulation is the inherent ability of the brain circulation to maintain constant cerebral blood flow over wide ranges of systemic blood pressure [17]. The basic pathomechanism of cerebral autoregulation may be described by the following equation:

$$ \mathrm{cerebral}\ \mathrm{blood}\ \mathrm{flow}=\frac{\mathrm{cerebral}\ \mathrm{perfusion}\ \mathrm{pressure}}{\mathrm{cerebrovascular}\ \mathrm{resistance}} $$

Assuming that cerebral perfusion pressure is proportional to the mean arterial pressure, if MAP decreases, a vasodilation of the resistance arterioles should occur in order to maintain a constant cerebral blood flow. However, in the present cohort of septic animals an increased pulsatility index was detected in the middle cerebral arteries. An increased pulsatility index suggest an increased resistance distal to the site of insonation [18]. Thus, cerebral arterioles became constricted during the development of sepsis. Additionally, we could demonstrate a significant relationship between percentual decreases in mean arterial pressure and percentual increases in pulsatility indices, which suggests that increased pulsatility index is proportional to the magnitude of mean arterial pressure decrease.

What is the possible explanation of these results? When cerebral hemodynamic data are analyzed together with other hemodynamic parameters gathered from the PiCCo measurements, it becomes obvious that the developing shock resulted in a decreased cardiac index and an increased systemic vascular resistance in septic animals. These observations are in accordance with the results of previous porcine experiments using E. coli [19–22]. Similar to our observations, Pranskunas and co-workers reported on a 2-fold increase of the systemic vascular resistance 5 h after injection of E.coli, accompanied by a gradual decrease of the cardiac index [20]. In a recent review models using continuous infusion of living bacteria are described as hypodynamic sepsis models [23]. Cerebral blood flow and cerebral autoregulation was so far not tested in hypodynamic sepsis.

According to our results, there is a strong vasoconstrictor activity present causing an increased SVRI together with an increased pulsatility index in the cerebral arteries. It is conceivable that although there might be an autoregulatory vasodilation of the resistance arterioles evoked by the decreased MAP/CPP, in this early phase of experimental shock a more potent vasoconstrictor activity is present that overwhelms autoregulatory vasodilation. Taking into consideration that resistance arterioles (~200 μm in diameter) are also the actors of autoregulatory response and metabolic regulation, the results of autoregulatory and metabolic regulatory tests may vary according to the actual phase of the developing septic process.

It has been demonstrated in previous studies that sepsis-related cerebral microcirculation alterations are characterized by a lower density of perfused capillaries, which can be related to elevated cerebrovascular resistance [24]. An increased distance between brain capillaries and astrocytes may result in unsatisfactory oxygen supply. High cerebrovascular resistance and disturbed cerebral autoregulation may expose septic patients to a decreased CBF if a compensatory elevation in CPP is absent. In an experimental study it was demonstrated that 18 h following the onset of sepsis cerebral hypoxia were registered only in animals with 65 mmHg of MAP or less, although they had similar density of functional cerebral capillaries and proportions of small perfused cerebral vessels compared to subjects with higher MAP values [25]. The main reason for the microcirculatory disturbances is the inhibition of eNOS enzyme by circulating cytokines (TNF-α, IFN-γ, IL-1 and IL-8), which causes a decreased NO production and thus vasoconstriction, deteriorating blood flow. Another possible cause is that the self-inducing inflammatory process and cytokine-storm disturb the balance of the pro- and anti-thrombotic system, and reduces the concentration of protein-C, thus the amount of activated protein-C (APC) level as well. In addition, the dysfunction of vascular endothelial cells also contributes to the disease and propagates the formation of edema- associated cerebral inflammation [26].

This may explain that cerebral blood flow has been documented to be low, normal or increased in experimental or human sepsis. The results of autoregulatory tests have also yielded variable results: human observations of Matta and Stow could not demonstrate autoregulation disturbance in septic patients [13]. Conversely, using transcranial Doppler sonography, both Smith and colleagues [27] as well as Pfister and colleagues [28] demonstrated affected autoregulatory responses in severe phases of sepsis. Similar to autoregulation tests, metabolic regulation tests (CO_2_-reactivity or acetazolamide) have reported on conflicting results [14–16, 29]. In the present study we could demonstrate a slight (but statistically not significant) decrease in the middle cerebral artery mean blood flow velocity along with an increase in pulsatility index. This may refer to cerebral arteriolar vasoconstriction in septic animals in the fulminant phase of experimental sepsis, which, however, cannot be generalized to all phases of the septic process. It has to be noted that although PI index is widely accepted as a descriptor of cerebrovascular resistance [30] a recent study indicated that in certain conditions –especially in gradually raised intracranial pressure with an exhausted autoregulatory reserve- TCD PI may be positively or negatively correlated with CVR. Therefore changes of the pulsatility index should be interpreted with caution [31]. In fact, intracranial pressure measurements were not performed in the present study, but in the animals we could not observe any signs of raised intracranial pressure (elevation of blood pressure, bradycardia) and thus the influencing effect of ICP was considered minimal. Another factor that theoretically could influence vasoreactivity during course of our measurements was the slight, but statistically significant decrease of pH. However, acidosis is a vasodilatory stimulus at the level of the cerebral arterioles that would rather lead to decrease in PI. [30]. Finally, it is known that anesthetic agents may also influence cerebral blood flow and cerebral metabolic rate for oxygen. We used ketamine and xylazine in combination in our animals. It is known that ketamine increases global and regional CBF through a calcium-dependent vasodilation and xylazine does not have any vascular effects in clinical doses [32]. They were used both in the control and the septic group, therefore their effect on the hemodynamic changes observed in septic animals could be excluded.

There are several limitations of the present study: We used a porcine model of intravenously administered E.coli suspension [20, 33]. Although this model is regarded as an endotoxicosis model rather than a classical sepsis model, it is widely accepted that it is suitable for modeling extreme clinical sepsis, such us meningococcaemia or gram-negative bacteremia in cases of granulocytopenia [34]*.* In a recent review article bacterial infusion model is proposed for better understanding of the pathophysiological mechanisms of sepsis [23]. The results should be interpreted with caution as results gathered from animal models may not be verifiable in humans, since animals are usually young and healthy in contrast to patients with sepsis who represent heterogenous cohort of critical illnesses with wide co-morbidities and concommittant medications. The second limitation of the work is that we did not use fluid resuscitation therapy and therefore only the early shock phase could be assessed because of the death of the septic animals. Therefore, our systemic and cerebral hemodynamic measurements provide pathophysiological information only on the early, fulminant phase of the development of septic shock.

## Conclusions

In conclusion, vasoconstriction occurs at the level of the cerebral arterioles, which overwhelmes autoregulatory response in hypodynamic experimental sepsis. These results may serve as additional pathophysiological information on the cerebral hemodynamic changes occurring during the septic process and may contribute to a better understanding of the pathomechanism of septic encephalopathy.

## Abbreviations

CBF: Cerebral blood flow
SVRI: Systemic vascular resistance
SVV: Stroke volume variation
TCD: Transcranial Doppler
